# *Ligustrazine hydrochloride* Prevents Ferroptosis by Activating the NRF2 Signaling Pathway in a High-Altitude Cerebral Edema Rat Model

**DOI:** 10.3390/ijms26031110

**Published:** 2025-01-27

**Authors:** Yue Han, Wenting Li, Huxinyue Duan, Nan Jia, Junling Liu, Hongying Zhang, Wenqian Song, Meihui Li, Yang He, Chunjie Wu, Yacong He

**Affiliations:** 1 State Key Laboratory of Southwestern Chinese Medicine Resources, School of Pharmacy, Chengdu University of Traditional Chinese Medicine, Chengdu 611137, China; hanyue@stu.cdutcm.edu.cn (Y.H.); duanhxy@stu.cdutcm.edu.cn (H.D.); zhonghongying@stu.cdutcm.edu.cn (H.Z.); 15184317449@163.com (W.S.); 19960797451@163.com (M.L.); 19915977825@163.com (Y.H.); 2 Department of Pharmacy, The Eighth Clinical College, Sun Yat-sen University, No. 3025, Shennan Middle Rd., Futian District, Shenzhen 518033, China; liwenting1111@126.com; 3 School of Clinical Medicine, Chengdu University of Traditional Chinese Medicine, Chengdu 610075, China; jianan0103@stu.cdutcm.edu.cn (N.J.); liujunling@stu.cdutcm.edu.cn (J.L.); 4 Innovative Institute of Chinese Medicine and Pharmacy, Academy for Interdiscipline, Chengdu University of Traditional Chinese Medicine, Chengdu 611137, China

**Keywords:** *Ligustrazine hydrochloride*, high-altitude cerebral edema, oxidative stress, ferroptosis, NRF2 signaling pathway

## Abstract

High-altitude cerebral edema (HACE) is a disorder caused by low pressure and hypoxia at high altitudes. Nevertheless, as of now, there is still a scarcity of safe and effective prevention and treatment methods. The active component of *Ligusticum* Chuanxiong, namely *Ligustrazine hydrochloride* (LH), has shown potential in the prevention and treatment of HACE due to its anti-inflammatory, antioxidant, and neuroprotective effects in nervous system disorders. Consequently, the potential protective effect of LH on HACE and its mechanism still need to be further explored. Prior to modeling, 90 male Sprague-Dawley rats were pretreated with different doses of drugs, including LH (100 mg/kg and 50 mg/kg), dexamethasone (4 mg/kg), and ML385 (30 mg/kg). Subsequently, the pretreated rats were placed in a low-pressure anoxic chamber simulating a plateau environment to establish the rat HACE model. The effects and mechanisms of LH on HACE rats were further elucidated through determination of brain water content, HE staining, ELISA, immunofluorescence, molecular docking, molecular dynamics simulation, western blot, and other techniques. The results showed, first of all, that LH pretreatment can effectively reduce brain water content; down-regulate the expression of AQP4, HIF-1α, and VEGF proteins; and alleviate damage to brain tissue and nerve cells. Secondly, compared with the HACE group, LH pretreatment can significantly reduce MDA levels and increase GSH and SOD levels. Additionally, LH decreased the levels of inflammatory factors IL-1β, IL-6, and TNF-α; reduced total iron content in brain tissue; increased the expression of ferroptosis-related proteins such as SLC7A11, GPX4, and FTH1; and alleviated ferroptosis occurrence. Molecular docking and molecular dynamics simulations show that LH has a strong binding affinity for NRF2 signaling. Western blot analysis further confirmed that LH promotes the translocation of NRF2 from the cytoplasm to the nucleus and activates the NRF2 signaling pathway to exert an antioxidant effect. The NRF2 inhibitor ML385 can reverse the anti-oxidative stress effect of LH and its protective effect on HACE rat brain tissue. In summary, LH may have a protective effect on HACE rats by activating the NRF2 signaling pathway, inhibiting ferroptosis, and resisting oxidative stress.

## 1. Introduction

High-altitude cerebral edema (HACE) is a significant neurological disorder impacting about 0.5% to 1.0% of individuals [1,2]. The disease mainly occurs in individuals who have not accustomed to the high-altitude environment and can be initiated when they ascend rapidly to high altitudes and are exposed to low pressure and hypoxia. High-altitude cerebral edema (HACE) frequently arises from acute mountain sickness (AMS) or high-altitude pulmonary edema (HAPE) [3]. Its clinical manifestations include symptoms like limb discoordination and decreased consciousness level, which may rapidly develop into coma [4,5,6]. If there is no timely treatment, HACE can lead to fatal outcomes within 24 h of its onset. Currently, there is no specific medicine to prevent the occurrence of HACE. Given the complex physiological and pathological processes of HACE [7], it is particularly important to explore its underlying mechanism and develop new therapeutic agents.

It is widely recognized that HACE primarily arises from changes in cellular metabolism and increased vascular permeability, caused by brain hypoxia in high-altitude conditions [8,9]. Oxidative stress primarily regulates these physiological changes, playing a crucial role in triggering subsequent chain reactions. At high altitudes, oxygen scarcity disrupts cellular energy metabolism, leading to anaerobic processes that elevate oxidative stress and produce oxidative by-products like malondialdehyde (MDA). These active substances have the ability to inflict damage on brain cell membranes and organelles, activating cell death mechanisms, leading to cell edema and dysfunction, and ultimately triggering HACE. Moreover, the rise of proinflammatory factors can also exacerbate the oxidative stress condition [10,11]. Oxidative stress disrupts cellular antioxidant defenses. This results in the depletion of the antioxidant glutathione (GSH) in lipid peroxides and hydroperoxides, leading to glutathione peroxidase 4 (GPX4) inactivation and subsequent ferroptosis [12].

Ferroptosis, an iron-dependent programmed cell death, is triggered by the inhibition of glutathione peroxidase 4 (GPX4) [13]. The procedure involves lipid peroxidation and iron metabolism imbalance, which damage the cell membrane structure and induce cell death [13,14,15]. As the prominent intracellular glutathione peroxidase capable of reducing lipid peroxides, GPX4 safeguards cells from damage by converting the peroxide bonds in lipid peroxides into hydroxyl groups and eliminating their peroxide activity. During ferroptosis, GPX4 expression is notably decreased, leading to higher lipid peroxidation and cell death [16]. Studies have manifested that ferroptosis acts as the core mechanism underlying hypoxic-ischemic brain damage [17]. Hypoxia and ischemia reduce SLC7A11 and GPX4 levels in brain tissue, resulting in brain damage [18,19]. Furthermore, past research has suggested a strong connection between the mechanism of ferroptosis and the onset of acute mountain sickness [20].

It deserves to be mentioned that NRF2 is not just an antioxidant factor but also plays a key role in the onset and progression of several cancerous and non-cancerous diseases [21,22]. NRF2, an important transcription factor, plays a vital role in antioxidant responses and is seen as a key regulator of ferroptosis. As a downstream gene of NRF2, GPX4 expression is heightened by increased NRF2 protein activity, which prevents lipid peroxidation and disrupts iron metabolism at the molecular level, ultimately achieving the objective of inhibiting ferroptosis. Activation of the NRF2 signaling pathway effectively inhibits ferroptosis [23]. Activating the NRF2 signaling pathway to regulate ferroptosis is considered a promising strategy for HACE treatment.

Screening safe and effective herbal monomers in natural medicines constitutes an important method for the research of potential drugs for HACE. *Ligusticum* chuanxiong (chuanxiong), a traditional Chinese medicinal herb, possesses pharmacological actions like promoting blood circulation, eliminating blood stasis, facilitating qi movement, alleviating pain, and dispelling wind along with eliminating dampness. The rhizome of chuanxiong is extensively utilized in traditional medicine for addressing headaches. Previous researchers have found through web-based pharmacological predictions that chuanxiong can help prevent diseases such as hypoxic-ischemic encephalopathy and ischemic stroke [24]. *Ligustrazine hydrochloride* (LH) is the principal active component in chuanxiong and has been demonstrated to possess substantial antioxidant activity and upregulate NRF2 expression, thereby defending against cerebral ischemia in rats [25]. In addition, it can inhibit apoptosis by reducing oxidative stress and protect cells from damage by inhibiting inflammation and reducing iron overload [26]. Additionally, it was identified that LH could safeguard neurons against death induced by hypoxia [27]. These studies indicate that LH could be highly effective in preventing and treating high-altitude cerebral edema, which is caused by low-pressure and low-oxygen conditions at high altitude.

Based on the context, we proposed that LH could pharmacologically prevent and treat HACE by potentially inhibiting ferroptosis through the NRF2 signaling pathway. To test this hypothesis, we built a rat HACE model and evaluated LH’s efficacy by measuring brain water content, conducting histopathological analysis, and assessing oxidative stress and inflammatory factor levels. Molecular docking, dynamics simulation, and western blot analyses were employed to elucidate the molecular mechanisms of HACE, providing novel scientific foundations and targets for its prevention and treatment. This study contributes to advancements in plateau medicine.

## 2. Results

### 2.1. Effect of LH Pre-Administration on HACE Model

**Figure 1:**
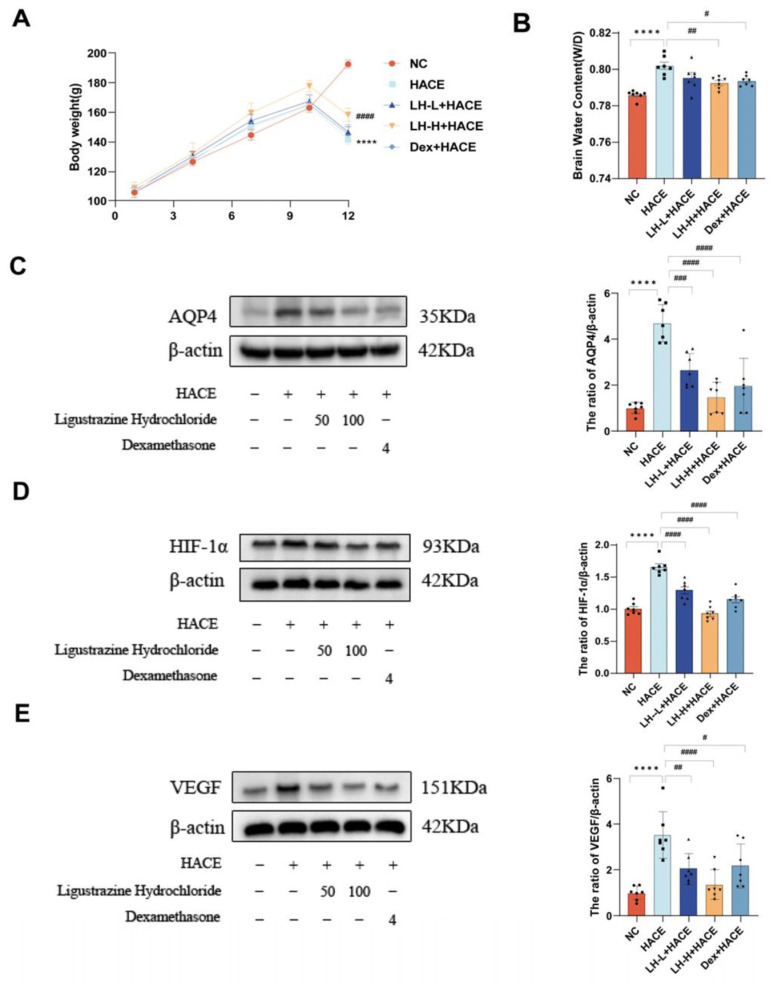
Effect of LH pre-administration on HACE model. (**A**) Weight modifications in every group. (**B**) Water content in the brain for each group. (**C**–**E**) Western blot analysis of HACE-related protein expression (left) and quantitative analysis (right). The values are presented as mean ± SEM (n = 7). **** p < 0.0001 when compared to the NC group; *^#^ p* < 0.1, *^##^ p* < 0.01, *^###^ p* < 0.001, and *^####^ p* < 0.0001 when compared to the HACE group.

The whole experimental process mainly included the following four periods: adaptive feeding (days 0–7), pre-administration (days 8–9), modeling (days 10–12), and sampling (days 12). During the whole experiment, the physiological parameters and body weight of the rats were systematically monitored and meticulously recorded. In the adaptive feeding and pre-administration stages, the mental state, sleep, breathing, eating, drinking, and excretion of the rats in each group were all within the normal range, and their body weight gradually increased. However, during the modeling phase, we observed that the experimental rats exhibited drowsiness, dyspnea, and a marked reduction in appetite and weight. Figure 1A illustrates that rat weights varied over time across all groups, with no significant differences observed on days 1, 4, 7, and 10. On the 12th day, the rats in the NC group maintained normal body weight, whereas the rats in the other groups exhibited varying degrees of weight loss. The HACE group rats exhibited significantly lower body weight compared to the NC group. In contrast, the LH pretreatment group showed improved physiological status and body weight relative to the HACE group, with the effect becoming more pronounced at higher doses. In conclusion, exposure to a low-pressure, hypoxic environment significantly impacts the physiological state and body weight of rats, while LH pretreatment can mitigate these effects.

Furthermore, we evaluated the extent of brain edema in the rats by quantifying the water content in brain tissue. In the HACE group, brain water content was significantly higher than in the NC group. As illustrated in Figure 1B, the brain water content in both the LH and dexamethasone pretreatment groups exhibited varying degrees of reduction. In addition, AQP4 is one of the aquaporins that may cause cerebral edema. There is evidence that the way AQP4 is expressed or regulated is closely associated with brain edema [28]. In this experiment, western blot analysis revealed that AQP4 expression was markedly elevated in the HACE group, but LH pretreatment significantly suppressed this expression.

In hypoxic environments, HIF-1α is regarded as a primary regulator of oxygen equilibrium and is activated [29]. VEGF, as one of the transcriptional targets of HIF-1α, is crucial for angiogenesis, regulation of vascular permeability, and promotion of new blood vessel formation, resulting in increased membrane permeability and capillaries under hypoxia conditions [30]. As shown in Figure 1C,D,E, according to western blot analysis, the HACE group exhibited upregulation of HIF-1α and VEGF, whereas the LH group showed a certain level of downregulation after being pretreated with LH. These results demonstrate that LH preconditioning could markedly diminish hypoxia and cerebral swelling in rats experiencing HACE.

### 2.2. LH Protected the Structure of Brain Tissue and Neurons in the HACE Model

**Figure 2:**
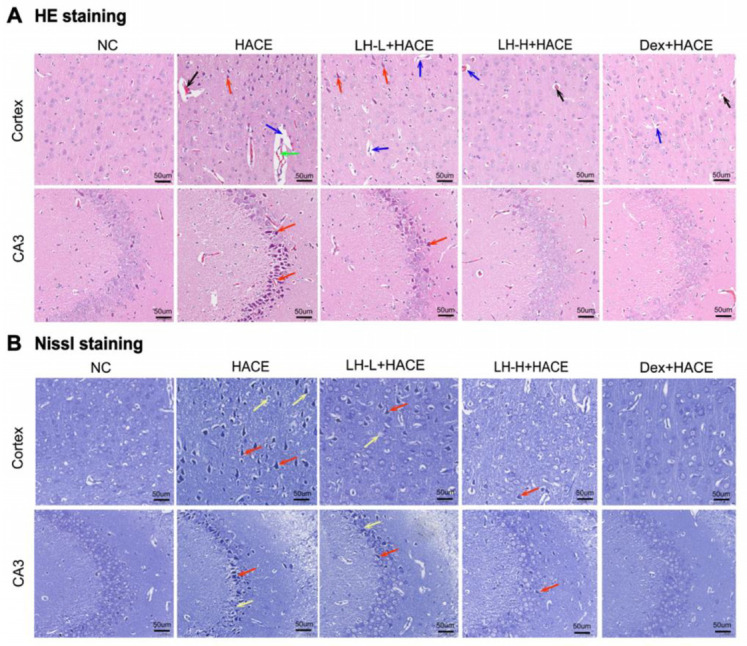
LH protected the structure of brain tissue and neurons in the HACE model. Representative photomicrographs illustrating morphological changes in brain tissues using HE staining (**A**) and Nissl staining (**B**). Bar = 50 um, magnification 20× (“→” black for vascular congestion; blue for widening of perivascular space; green for vasodilation; red for crumpling of neuronal cells; yellow for lysis of nuclear membrane or nucleoli).

In the HACE model rats, HE and Nissl staining were used to assess the impact of LH on pathological alterations and neuronal integrity in the cerebral cortex and hippocampus. HE staining showed that neurons in the CA3 region of the hippocampus and the cortical area of the control group were plentiful and orderly, without morphological damage or capillary congestion. The HACE group exhibited significant expansion in the CA3 region of the hippocampus and the cortical perivascular spaces compared to the NC group. Additionally, cellular organization was markedly disordered and sparse. However, pre-administration of LH markedly ameliorated cerebrovascular edema and vascular space enlargement, particularly at the 100 mg/kg dose (Figure 2A).

As illustrated in Figure 2B, Nissl staining results showed that neurons in the NC group were well-structured, containing distinct blue granular vesicles with clearly visible nucleoli and nuclear membranes. However, neurons in the HACE group showed abnormal shapes, decreased survival, and nuclear membranes and nucleoli that are unclear or dissolved. In contrast, the LH pretreatment group exhibited a notable reduction in these pathological changes. It can be seen that hypobaric hypoxia can induce severe pathological damage to brain tissue, while LH preconditioning exerts a beneficial effect on mitigating such damage and safeguarding the morphological integrity of neuronal structures and brain tissue.

### 2.3. LH Alleviates Inflammation in HACE Model

**Figure 3:**
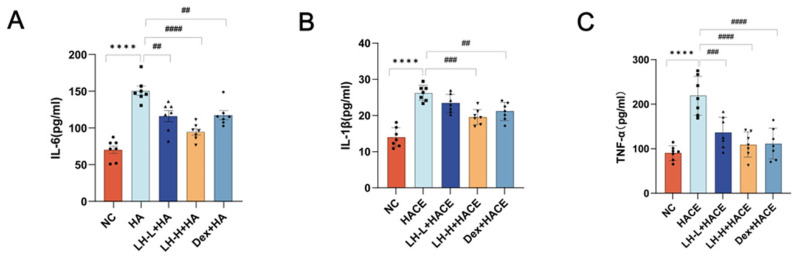
LH alleviates inflammation in HACE model. (**A**–**C**) Serum levels of IL-1β, TNF-α, and IL-6 were measured across all groups. Values are expressed as mean ± SEM for *n* = 7. **** p < 0.0001 vs. NC group; *^##^ p* < 0.01, *^###^ p* < 0.001, *^####^ p* < 0.0001 vs. HACE group.

Under hypoxic conditions, the production and accumulation of inflammatory cytokines exacerbate vascular endothelial cell injury [31]. Clinical studies conducted previously have demonstrated that high levels of circulating pro-inflammatory cytokines can result in edema in the lungs or brain. The progression of HACE is marked by elevated levels of serum proinflammatory cytokines IL-1β, TNF-α, and IL-6 [32]. The study found that post-exposure, the HACE group exhibited significantly elevated serum levels of IL-6, IL-1β, and TNF-α compared to the NC group, indicating that proinflammatory agents induced by hypobaric hypoxia may contribute to brain injury. The LH treatment groups exhibited significantly reduced levels of IL-6, IL-1β, and TNF-α compared to the HACE group, aligning more closely with the NC group levels (Figure 3). LH pretreatment significantly inhibited the release of proinflammatory factors induced by hypobaric hypoxia. These findings indicate that LH preconditioning reduces inflammatory responses in HACE rats, thereby mitigating brain injury.

### 2.4. LH Reduced Oxidative Stress in the HACE Model

**Figure 4:**
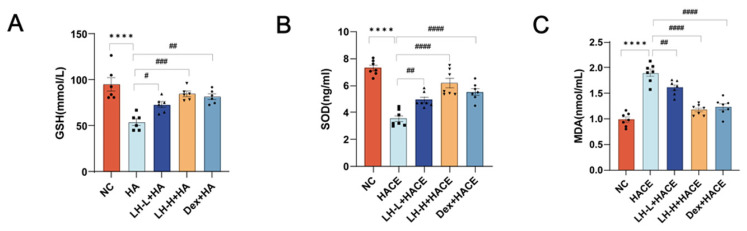
LH reduced oxidative stress in the HACE model. (**A**–**C**) The blood serum levels of GSH, SOD, and MDA for each group. Values are expressed as mean ± SEM for *n* = 7. **** *p* < 0.0001 vs. NC group; ^#^ *p* < 0.1, ^##^ *p* < 0.01, ^###^ *p* < 0.001, ^####^ *p* < 0.0001 vs. HACE group.

Past investigations have indicated that acute oxygen deficiency can cause heightened oxidative stress and resulting damage [33]. Oxidative stress can elevate the consumption of GSH and SOD, resulting in reduced activity or levels. Elevated MDA expression levels serve as an indicator of oxidative stress. To verify the protective effects of LH on HACE rats, we employed ELISA assays to investigate whether LH can attenuate hypoxia-induced oxidative stress. Figure 4 illustrates the evaluation of serum GSH, SOD, and MDA concentrations in rats to assess LH’s antioxidant activity. HACE rats exhibited significantly elevated serum MDA levels and reduced GSH and SOD levels compared to the NC group. Both the LH-L + HACE and LH-H + HACE groups demonstrated a decrease in MDA levels and an increase in GSH levels to some extent, implying that LH may halt the pathological evolution of HACE by reducing oxidative stress.

### 2.5. LH Prevented Ferroptosis in HACE Rats

**Figure 5:**
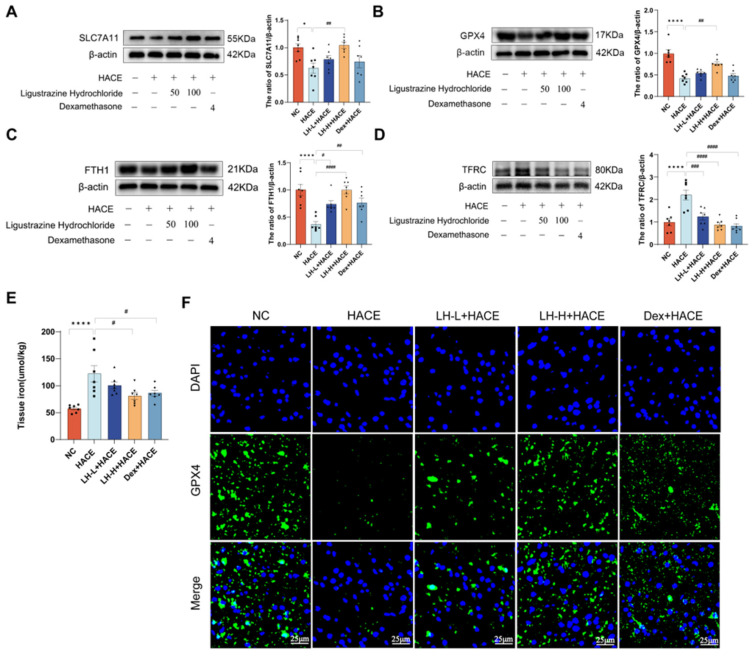
LH prevented ferroptosis in HACE rats. (**A**–**D**) Western blot analysis (left) and quantitative analysis (right) of the expressions of ferroptosis-related proteins. (**E**) Tissue iron in each group. (**F**) GPX4 representative image of immunofluorescence section of brain tissue (magnification 100×, scale = 25 μm). Values are expressed as mean ± SEM for *n* = 7. Significance levels are indicated as follows: **** *p* < 0.0001, * *p* < 0.1 compared to the NC group; ^#^ *p* < 0.1, ^##^ *p* < 0.01, ^###^ *p* < 0.001, ^####^ *p* < 0.0001 compared to the HACE group.

Researchers used western blotting to evaluate the levels of ferroptosis-related proteins, such as SLC7A11, GPX4, FTH1, and TFRC, in brain tissue to investigate the impact of LH on ferroptosis. Figure 5A–D illustrates that the HACE group exhibited reduced expression levels of SLC7A11, FTH1, and GPX4, while the TFRC group showed increased levels, in comparison to the NC group. LH pretreatment inhibited the expression of a ferroptosis-related protein. In addition, we studied iron levels in brain tissue to assess potential brain damage associated with ferroptosis. After exposure to hypobaric hypoxia, iron levels in brain tissue rose in the HACE group, whereas they significantly dropped in the LH and Dex pretreatment groups (Figure 5E).

GPX4 is crucial in inhibiting ferroptosis, thereby preventing cell death through this pathway [34]. Therefore, immunofluorescence staining was used to determine the occurrence of ferroptosis again. As shown in Figure 5F, consistent with the results of western blotting, GPX4 expression in the HACE group was dramatically reduced, while GPX4 levels increased following LH administration. These findings suggest that LH preconditioning may alleviate high-altitude cerebral edema by reducing ferroptosis.

### 2.6. Potential Regulation of LH Towards the NRF2 Signaling Pathway

**Figure 6:**
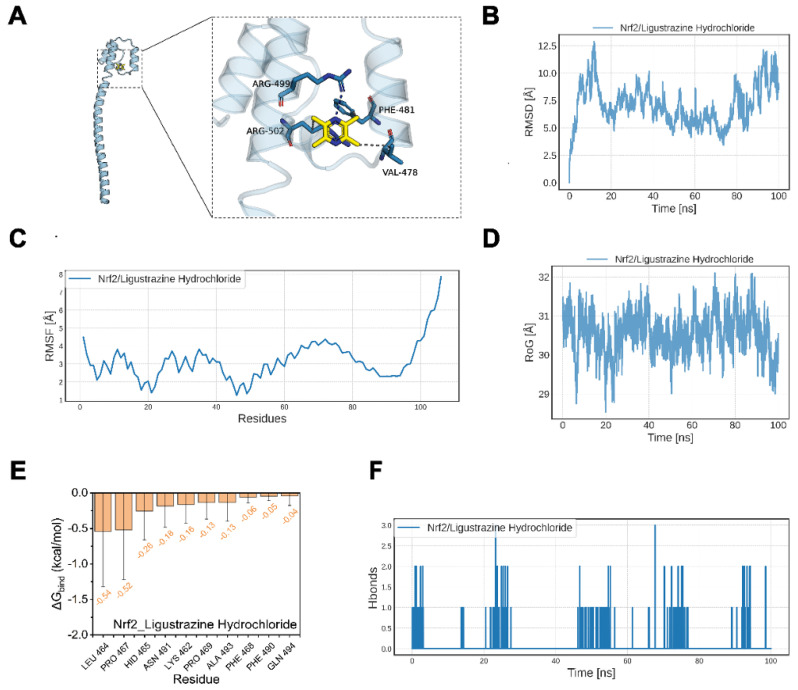
Potential regulation of LH towards the NRF2 signaling pathway. (**A**) NRF2 and LH molecular docking analysis. According to molecular docking studies, the binding mechanism is shown in general on the left and in detail on the right. (**B**) The RMSD of the complexes over time during the molecular dynamics simulation. (**C**) The RMSF was calculated from the molecular dynamics simulation trajectory. (**D**) The molecular dynamics simulation trajectory is analyzed using the radius of RoG. (**E**) The top ten amino acids that aid in protein and small molecule binding. (**F**) The number of hydrogen bonds between proteins and small molecules changes over the course of the molecular dynamics simulation.

Molecular docking and dynamics simulations were employed to investigate the interaction between LH and the NRF2 kinase domain, aiming to elucidate LH’s potential mechanism on HACE. Figure 6A displays the three-dimensional docking data of the NRF2 protein and LH. In the interaction with NRF2 protein, LH demonstrates significant hydrophobic interactions with ASP-661, ILE-659, and VAL-667 and forms a stable hydrogen bond with ARG-499. The affinity score for LH binding to the NRF2 protein was −4.083 kcal/mol, as indicated in Table 1, suggesting that the kinase domain of NRF2 may interact with LH.

Figure 6B,C illustrates how the RMSD of the NRF2/LH complex varies between 3 and 12 Å and the RMSF value falls between 1.5 and 7.0 Å in molecular dynamics simulation. In particular, there were significant dynamic changes in the 50~100 residues and terminal regions, which all reflected the greater flexibility and structural instability of NRF2 protein itself, which may reduce the binding stability of LH to NRF2, indicating that NRF2 protein has higher dynamic and weak binding adaptability. In order to further verify the flexibility of NRF2 protein, in the radius of gyration (RoG) analysis, the RoG value of the NRF2/LH complex was maintained at a high level of about 30 Å, accompanied by certain fluctuations, which may affect its binding stability and binding mode with LH (Figure 6D).

The MM-GBSA method was employed to evaluate the ligand-protein binding free energy from molecular dynamics simulation trajectories, enhancing the assessment of the interaction between LH and NRF2. According to the findings in Table 2, LH has a considerable capacity to bind with the NRF2 protein, as evidenced by the binding free energy of NRF2/LH being −3.73 ± 2.13 kcal/mol. MM-GBSA energy decomposition can identify the top 10 amino acid residues in the NRF2 protein most influential in LH binding, highlighting the key residues in the LH–NRF2 interaction, as depicted in Figure 6E. During the simulation, Figure 6F illustrates that the NRF2/LH complex predominantly formed 0–2 hydrogen bonds with a high frequency yet lacked persistent strong binding. This suggests that the interaction between LH and NRF2 proteins is likely driven by hydrophobic interactions or transient hydrogen bonding. Integrating molecular docking and dynamics simulations indicates that LH exhibits a strong affinity for the NRF2 kinase domain, implying its potential to form a stable bond with NRF2 and activate the NRF2 signaling pathway.

### 2.7. LH Activates the NRF2 Signaling Pathway in the HACE Model

**Figure 7:**
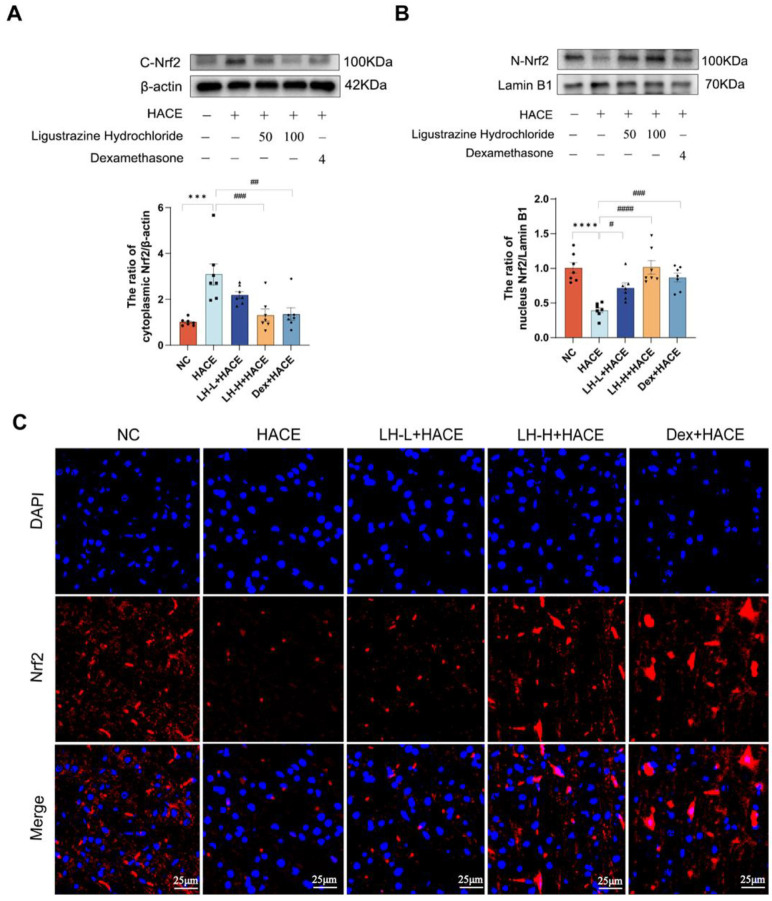
LH activates the NRF2 signaling pathway in the HACE model. (**A**,**B**) NRF2 expressions were analyzed quantitatively (bottom) and by western blot (top); (**C**) A representative image of the NRF2 immunofluorescence section of brain tissue (scale = 25 μm, magnification 100×). The mean ± SEM is used to express the values (*n* = 7). Significant differences were observed when compared to the NC group (**** *p* < 0.0001, *** *p* < 0.001) and the HACE group (^####^ *p* < 0.0001, ^###^ *p* < 0.001, ^##^ *p* < 0.01, ^#^ *p* < 0.1).

Based on prior molecular docking and dynamics simulations, we examined LH’s influence on the nuclear translocation of NRF2 protein in HACE model rats. Using the western blot test, we first determined the expression level of NRF2 in the cytoplasm and nucleus of the rat brain. According to the findings, exposure to hypoxia considerably raised the amount of NRF2 expression in the cytoplasm. NRF2 expression was upregulated in the nucleus and dramatically reduced in the cytoplasm following LH pretreatment (Figure 7A,B). This indicates that LH promotes the translocation of NRF2 from the cytoplasm to the nucleus, facilitating its antioxidant function. Furthermore, as illustrated in Figure 7C, immunofluorescence results demonstrated that, in contrast to the NC group, NRF2 expression was significantly lower in the HACE group and progressively upregulated in the LH pretreatment group as the dose increased.

### 2.8. LH’s Ability to Combat Oxidative Stress Relies on the NRF2 Signaling Pathway

**Figure 8:**
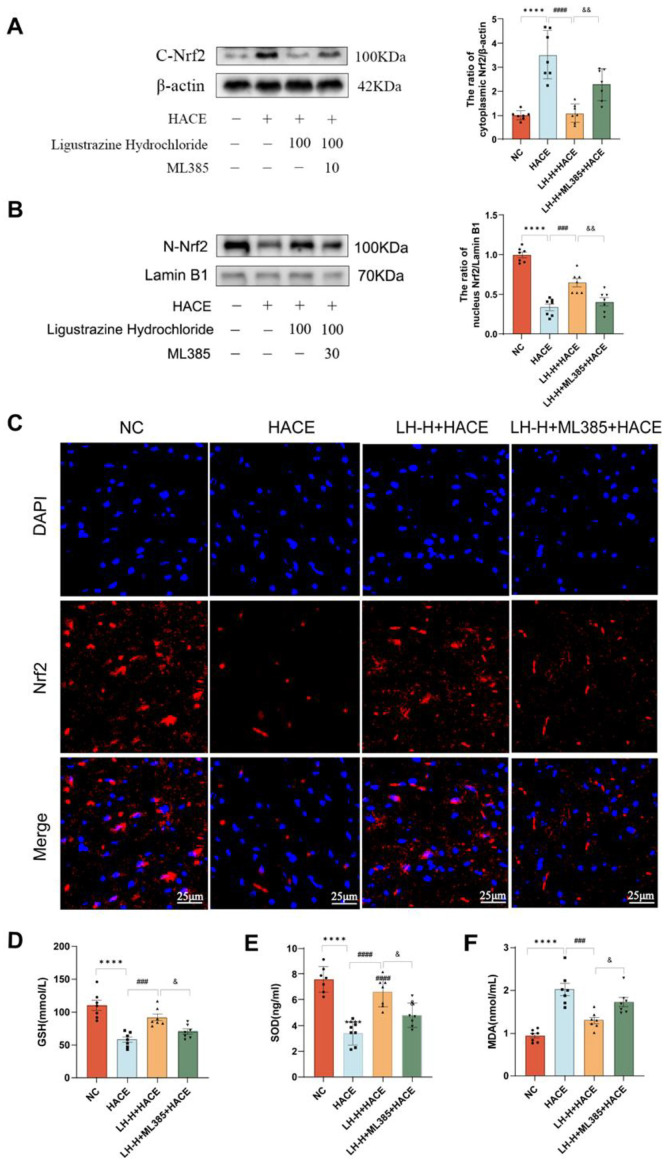
LH’s ability to combat oxidative stress relies on the NRF2 signaling pathway. (**A**,**B**) Western blot analysis (left) and quantitative analysis (right) of NRF2 expression. (**C**) Representative picture of NRF2 in an immunofluorescence segment of brain tissue (magnification 400×, scale = 100 μm). (**D**–**F**) GSH, SOD, and MDA levels in blood serum for each group. Results are expressed as mean ± SEM with a sample size of 7. Significance levels are indicated as follows: **** *p* < 0.0001 when compared to the NC group; ^###^ *p* < 0.001, ^####^ *p* < 0.0001 when compared to the HACE group; and ^&^ *p* < 0.1, ^&&^ *p* < 0.01 when compared to the LH-H + HACE group.

We investigated whether the anti-oxidative stress effect of LH in the HACE rat model depends on the activation of NRF2 signaling pathway. As shown in Figure 8A,B, NRF2 in the LH-H + HACE group is activated to promote the transfer of NRF2 from cytoplasm to nucleus, binds to the antioxidant reaction elements in the nucleus, and starts the transcription expression of genes for antioxidant enzymes, thereby reducing oxidative stress and protecting cells from damage [35]. ML385 inhibited the activity of NRF2 and decreased the occurrence of nuclear translocation. In the meantime, immunofluorescence was displayed in Figure 8C, with the LH-H + HACE group exhibiting a markedly higher fluorescence intensity than the HACE group, however, ML385 counteracted this effect. Finally, the changes in oxidative stress indexes were verified. LH pretreatment dramatically reduced MDA activity and increased GSH and SOD expression levels in the HACE model. However, the changes in GSH, SOD, and MDA levels brought on by LH were counteracted by ML385. These findings suggest that LH facilitates the nuclear translocation and activation of the NRF2 signaling pathway to exert its anti-oxidative stress effects (Figure 8D–F).

### 2.9. LH Prevented Ferroptosis Through the NRF2 Signaling Pathway

**Figure 9:**
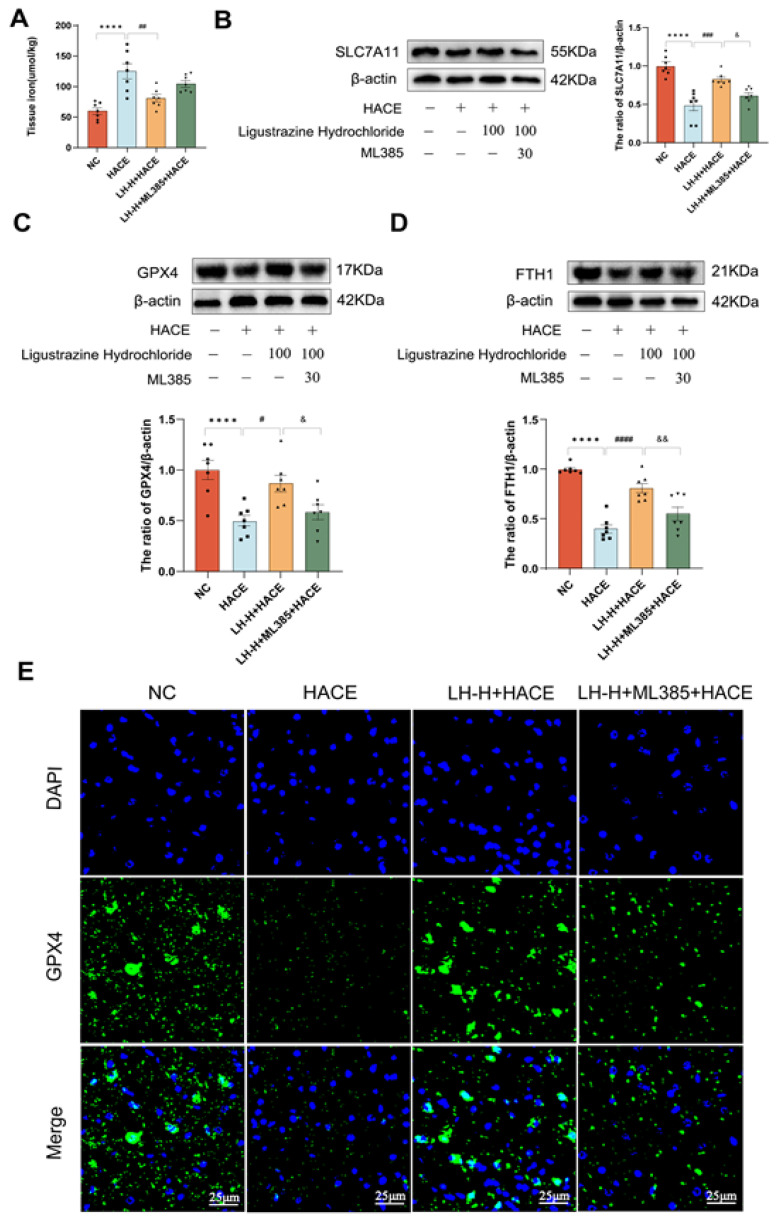
LH prevented ferroptosis through the NRF2 signaling pathway. (**A**) Tissue iron in each group. (**B**–**D**) Western blot analysis (left-top) and quantitative analysis (right-bottom) of ferroptosis-related protein expressions. (**E**) Representative picture of GPX4 in brain tissue immunofluorescence (magnification: 100×; scale: 25μm). Results are expressed as mean ± SEM with a sample size of 7. Significance levels are indicated as follows: **** *p* < 0.0001 when compared to the NC group; ^#^ *p* < 0.1, ^##^ *p* < 0.01, ^###^ *p* < 0.001, ^####^ *p* < 0.0001 when compared to the HACE group; and ^&^ *p* < 0.1, ^&&^ *p* < 0.01 when compared to the LH-H+HACE group.

To verify that LH mitigates ferroptosis through NRF2 pathway activation, we administered the NRF2 inhibitor ML385 to HACE rats for further investigation. Initially, the overall iron levels in the brain tissue were assessed. In Figure 9A, it is evident that the LH-H + HACE group experienced a significant drop in brain tissue iron content compared to the HACE group. However, ML385 reversed LH’s downregulation, leading to an increase in iron content in the LH + ML385 + HACE group. Furthermore, western blotting was employed to detect the levels of ferroptosis-related proteins, including SLC7A11, GPX4, and FTH1. Figure 9B–D illustrates that the LH-H + HACE group exhibited increased expression levels of SLC7A11, GPX4, and FTH1 compared to the HACE group. The LH-H + ML385 + HACE group exhibited a reduction in the protein expressions of SLC7A11, GPX4, and FTH1 compared to the LH-H + HACE group. These results indicated that ML385 intervention promoted the expression of ferroptosis-related proteins. This study utilized immunofluorescence staining of GPX4, a key ferroptosis marker, to detect ferroptosis. The fluorescence expression of GPX4 in the LH-H + HACE group was considerably higher than in the HACE group; however, it was lower in the LH-H + ML385 + HACE group. These findings imply that ML385 reversed the inhibitory impact of LH on ferroptosis (Figure 9E).

### 2.10. The Protective Effect of LH on HACE Depends on the NRF2 Signaling Pathway

**Figure 10:**
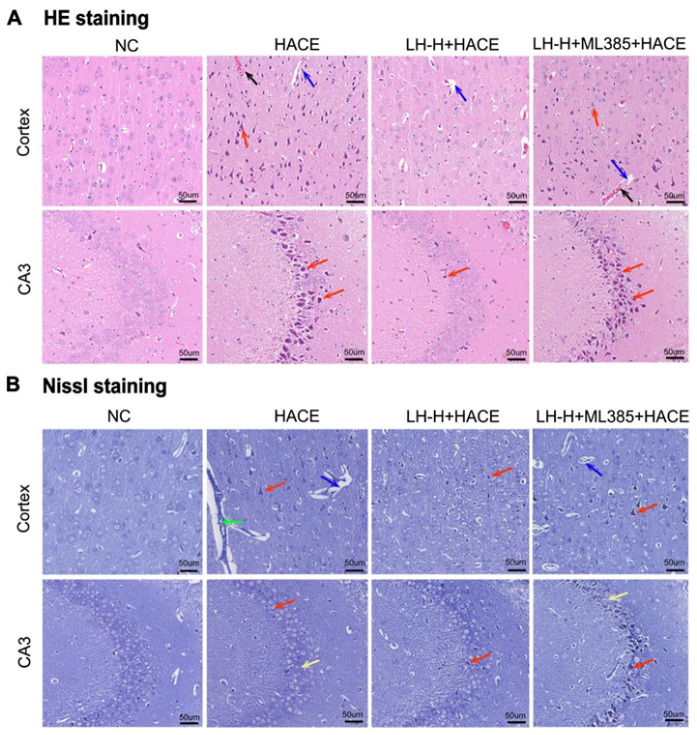
The protective effect of LH on HACE depends on the NRF2 Signaling Pathway. (**A**) Photomicrographs of brain tissue morphological changes using HE staining. (**B**) Photomicrographs of brain tissue morphological changes using Nissl staining. Bar = 50 μm, magnification: 20× (“→” black for vascular congestion; blue for widening of perivascular space; green for vasodilation; red for crumpling of neuronal cells; yellow for lysis of nuclear membrane or nucleoli).

To confirm the association between LH’s preventative effect on HACE generated by hypobaric hypoxia and the NRF2 signaling pathway, we employed HE and Nissl staining to examine the hippocampus and cortex. As shown in Figure 10A, LH pretreatment significantly improved the disorder of cell arrangement, reduced swelling and vasodilation in HACE group. Furthermore, Nissl staining revealed that LH preconditioning improved neuronal morphology and blurred nucleolar membranes (Figure 10B). ML385 counteracted these beneficial effects, suggesting that LH’s protective mechanism against HACE may involve activation of the NRF2 signaling pathway.

## 3. Discussion

With the expansion of human activities, the incidence of HACE is rising, highlighting the urgent need for the rapid development of distinct and effective therapeutic interventions. Recently, the active compounds in Chinese herbal medicine for preventing and treating HACE have garnered increased attention. This study is the first to demonstrate the protective effect of LH against acute hypobaric hypoxia-induced HACE in rats. This may be related to the fact that LH attenuates the onset of oxidative stress by activating the NRF2 pathway, thereby alleviating hypoxia-induced ferroptosis. Unlike the synthetic drug dexamethasone, LH is a natural substance with fewer side effects, making it a safer option. These findings suggest that LH could be a novel HACE agent.

The rapid exposure to low pressure and hypoxia at high altitude may lead to headaches, nausea, vomiting, and lack of appetite [36]. In the current investigation, we replicated a hypobaric hypoxic environment at 6000 m altitude for 48 h to generate a rat model of HACE. During the modeling procedure, we noticed that the model rats presented the above symptoms and significant weight loss, indicating that the low-pressure hypoxic environment had a significant effect on the physiological state of the rats. We assessed brain edema by measuring changes in BWC. The BWC was found to be considerably higher in HACE rats than in normal controls, providing direct evidence of brain edema. HE and Nissl staining showed that in the HACE group, the perivascular space was significantly wider, and the arrangement of neuronal cells was disordered and sparse. The above pathological alterations in the rats were in line with the autopsy results of HACE patients recorded in the literature [37], thus verifying the successful establishment of our experimental model. However, LH pretreatment significantly reduced the weight loss of HACE rats, improved the physiological condition of rats during modeling, decreased the BWC, and attenuated brain tissue damage. These results suggest that LH preconditioning might partially relieve the brain edema caused by the hypoxic environment at high altitudes in rats. In addition, HIF-1α is a key protein that regulates mammalian responses and transcriptional activity under hypoxic conditions [29]. VEGF, as one of the transcriptional targets of HIF-1α, significantly contributes to the severe vasogenic edema associated with HACE at high altitude [38]. AQP4 is required to maintain water balance in the brain, and higher AQP4 expression indicates the presence of water retention [39]. The study revealed significantly elevated protein expression levels of HIF-1α, AQP4, and VEGF in brain tissues of the hypobaric hypoxia exposure group compared to the control group. This indicates that the high-altitude environment could lead to hypoxia and edema. Nevertheless, in the LH preconditioning group, the aforementioned effects were reversed, and the expression of HIF-1α, AQP4, and VEGF was notably decreased, indicating that LH might possess anti-hypoxia and anti-edema effects in rats with HACE.

Inflammation is considered a contributing factor to cerebral edema, significantly impacting the neurological complications associated with HACE [40]. Hypoxia can induce the release of proinflammatory cytokines like IL-1β, IL-6, and TNF-α, which activate signaling pathways leading to an inflammatory response [41]. Our study validated the model’s success by identifying a notable rise in serum concentrations of IL-1β, IL-6, and TNF-α in HACE model rats. Pre-administration of LH notably decreased inflammatory factor levels, suggesting that LH mitigates brain damage in HACE rats by diminishing the inflammatory response.

Furthermore, attaining a high altitude can give rise to an acute condition of hypobaric hypoxia. The brain tissue is particularly susceptible to oxidative stress due to its high levels of unsaturated fatty acids and limited antioxidant enzymes [42]. Hypobaric and hypoxic conditions induce excessive reactive oxygen species production, leading to cell membrane lipid peroxidation. This procedure eventually produces detrimental compounds, among which is MDA [43]. MDA, a known biomarker for oxidative stress and lipid peroxidation, indicates cellular damage severity, with elevated levels marking the onset of oxidative stress in brain tissue [44]. Furthermore, GSH serves as a crucial antioxidant that alleviates oxidative stress by neutralizing free radicals and safeguarding cell integrity. SOD, a type of metalloproteinase, is primarily employed to transform superoxide anion free radicals into hydrogen peroxide and oxygen, thereby decreasing the quantity of superoxide anion and minimizing the harm to cells. Within the antioxidant system, GSH and SOD exhibit a synergistic and interdependent influence in eliminating free radicals within the body [45,46]. SOD first transforms superoxide anions into hydrogen peroxide, which is then converted into water and glutathione by GSH-Px using GSH, completing the antioxidant process and safeguarding cells from oxidative damage [47]. In the hypobaric and hypoxic environment, a dynamic equilibrium is established among GSH, SOD, and MDA. With the augmented consumption of GSH and SOD, they are capable of counteracting the increase of reactive oxygen species and preserving the redox balance within the cell. Oxidative stress can elevate the consumption of GSH and SOD, resulting in reduced activity or levels [48]. At this point, the content of MDA could be further elevated, reflecting the degree of intracellular lipid peroxidation and the continuous enhancement of oxidative stress levels. Our experimental findings showed that hypobaric hypoxia exposure increased MDA levels while decreasing GSH and SOD levels. On the contrary, a remarkable decrease in MDA levels was noticed in the LH-treated groups, along with a significant increase in GSH and SOD levels, indicating that LH provides a protective impact against hypobaric hypoxia-induced oxidative stress in HACE rats.

NRF2 is a key regulator of oxidative stress, modulating cellular redoxbalance and maintaining homeostasis [49]. It represents the core component of the cellular antioxidant defense system. Normally, NRF2 is found in the cytoplasm and forms a complex with the inhibitory protein KEAP1. This complex is promptly degraded through the proteasome pathway, thereby keeping the level of NRF2 within the cell low [50,51]. Nevertheless, when cells are subjected to oxidative stress or other adverse factors, NRF2 detaches from KEAP1 and gets activated. Activated NRF2 swiftly translocates to the nucleus and attaches to the ARE [52]. This binding enables NRF2 to initiate the transcription of genes for various antioxidant enzymes and antioxidants, including GPX4. NRF2 is an important upstream target of SLC7A11 and GPX4. When oxidative stress occurs, NRF2 is activated and translocated from the cytoplasm to the nucleus, resulting in increased expression of SLC7A11 and GPX4 and enhanced cellular antioxidant capacity. SLC7A11 and GPX4 are also involved in ferroptosis. The main causes of ferroptosis are GSH depletion and decrease in GPX4 activity. The GPX family is crucial in the process of ferroptosis [53]. GPX4 converts glutathione to GSSG and reduces L-OOH to L-OH. When cystine transport protein is blocked or GPX4 is inhibited, intracellular GSH is consumed, leading to GPX4 inactivation. GPX4-catalyzed glutathione reductase reactions are unable to metabolize lipid peroxides. Accumulation of lipid peroxides to a certain degree triggers ferroptosis [54,55]. SLC7A11 functions as a suppressor of ferroptosis and constitutes one of the most significant upstream regulators of ferroptosis [56]. SLC7A11 is an amino acid transporter and catalytic subunit of the Xc-system, which, together with SLC3A2, forms a system to transport cystine and convert it into cysteine for the synthesis of glutathione. SLC7A11 facilitates the production of reduced GSH, serving as a cofactor for GPX4, which transforms lipid hydroperoxides into less harmful substances, thereby mitigating oxidative stress, protecting cells from damage, and preventing ferroptosis [57]. Thus, the migration of NRF2 between the cytoplasm and the nucleus is one of the crucial mechanisms by which NRF2 regulates ferroptosis and anti-oxidative stress responses. Increasing the expression of GPX4 and SLC7A11 via the NRF2 signaling pathway increases cell antioxidant capacity, hence avoiding ferroptosis [58].

Oxidative stress induces lipid peroxidation, whereas hypoxia disrupts intracellular iron metabolism, resulting in the buildup of iron ions [59]. The accumulation of iron ions increases cellular sensitivity to oxidative stress and lipid peroxidation, triggering ferroptosis. Ferroptosis is an iron-dependent type of controlled cell death whose mechanism is primarily attributed to iron metabolism disorders and antioxidant system imbalances. FTH1 and TFRC serve as key biomarkers and regulators in iron metabolism [60]. The main role of FTH1 is to promote intracellular iron storage, thereby regulating iron absorption and release. It has been reported that silencing the FTH1 gene leads to a considerable decrease in superoxide dismutase activity and brain iron concentration, which initiates oxidative stress and disrupts iron homeostasis [61,62]. Transferrin is an iron-binding glycoprotein that transports iron to various tissues in the body by binding ferric iron (Fe^3+^) and interacting with TFRC through endocytosis [63]. Once the transferrin-TFRC complex is internalized, iron is liberated from TFRC and reduced to ferrous iron (Fe^2+^) in the cytoplasm, resulting in abnormal iron accumulation that may eventually cause ferroptosis and aggravate cell damage [54].

Our study showed that low-pressure hypoxia leads to oxidative stress, and when oxidative stress occurs, LH pre-treatment activates NRF2, which translocates from the cytoplasm to the nucleus and increases the expression of SLC7A11 and GPX4, which together exert antioxidant effects. In addition, compared with the NC group, the HACE group showed a significant increase in brain tissue iron content and TFRC protein expression and a significant decrease in FTH1 protein expression, whereas LH pretreatment significantly reduced brain iron content, increased FTH1 protein expression, and decreased TFRC protein content compared with the HACE group. Thus, the low-pressure hypoxic environment at high altitude triggers oxidative stress, which induces an imbalance in the antioxidant system and leads to the development of ferroptosis. These results suggest that LH may prevent the development of ferroptosis by activating the antioxidant system and controlling iron metabolism.

To further investigate whether the antioxidant activity and its inhibitory effects on LH ferroptosis were related to the control of the NRF2 signaling system, this study used ML385, a NRF2 signaling pathway inhibitor, for verification [64]. ML385 blocks NRF2 from binding to ARE by directly engaging with its DNA binding domain, resulting in downregulation of NRF2 target gene expression and, consequently, suppression of NRF2 signaling activation [65]. The study found that nuclear NRF2 expression was reduced in the group treated with both LH and ML385 compared to the LH-only group, indicating that ML385 effectively inhibited LH-induced activation of the NRF2 signaling pathway. The significant reduction in ferroptosis marker proteins GPX4, SLC7A11, and FTH1, along with increased iron content, elevated oxidative stress marker MDA, and decreased levels of SOD and GSH, suggests that LH’s inhibition of ferroptosis is connected to its antioxidant effects through the NRF2 signaling pathway. Finally, the addition of ML385 reversed the protective effect of LH on brain tissue and nerve cells in HACE rats, further confirming that the protective effect of LH on HACE depended on the NRF2 signaling pathway.

Our findings indicate that LH reduces oxidative stress and ferroptosis through the activation of the NRF2 pathway. Nonetheless, our research on the mechanism of LH action is still at the in vivo animal stage and requires further validation through additional experimental methods. Thus, our future plans include conducting in vivo knockout experiments in rats, performing immunoprecipitation analysis, and utilizing in vitro cellular models to delve deeper into the biological mechanisms underlying the interaction between LH and the NRF2 pathway. Additionally, the plateau environment is defined by its low pressure, lack of oxygen, cold temperatures, low humidity, and high levels of ultraviolet radiation. The low-pressure and hypoxic chamber we utilized was unable to completely replicate the plateau environment, making it essential to conduct research on the plateau field model when feasible.

## 4. Materials and Methods

### 4.1. Reagents and Antibodies

*Ligustrazine hydrochloride* (LH, A0189), having a purity of no less than 98%, was procured from Chengdu Munster Biotechnology Co., Ltd., located in Chengdu, China. ML385 (S8790) was sourced from Selleck Chemicals located in Houston, TX, USA. Primary antibodies against VEGF (AB32152) were purchased from Abcam (Cambridge, UK). AQP4 (68448-1-Ig) was purchased from Proteintech Group Co., Ltd. (Wuhan, China). NRF2 (WL02135) and HIF-1α (WL01607) were purchased from Wan Class Biotechnology Co., Ltd. (Shenyang, China). GPX4 (T56959S), SLC7A11 (T57046S), FTH1 (T55648F), and TFRC (T56618F) were purchased from Abmart Medical Technology Co., Ltd. (Shanghai, China). IL-6 (RX302856R), IL-1β (RX302869R), TNF-α (RX302058R), MDA (RXJ302836R), GSH (RXJ302694R), and SOD (RX301341R) were purchased from Ruixin Biotechnology Co., Ltd. (Quanzhou, China). The total iron ion assay kit (G4301) was purchased from Sevier Biotechnology Co., Ltd. (Wuhan, China).

### 4.2. Experimental Animals

Six-week-old male Sprague-Dawley rats, weighing 100–120 g, were obtained from Beijing Sibeifu Biotechnology Co., Ltd., with production certificate number SCXK (Jing) 2019-0010. The animals were kept under uniform laboratory conditions for 7 days (24 ± 1 °C, 55% ± 5% relative humidity, 12 h light/dark cycle) with unrestricted access to standard food and water.

This study was conducted in accordance with NIH guidelines for laboratory animal care and received approval from the Animal Care and Use Committee of Chengdu University of Traditional Chinese Medicine (Grant No. 2023386).

### 4.3. Experimental Design

#### 4.3.1. Groups and Animal Treatment

The experiment consisted of two phases. In the first phase, fifty SD rats were randomly divided into five groups of ten: the normal pressure normoxia control group (NC group), the low pressure hypoxia model group (HACE group), the *ligustrazine hydrochloride* 50 mg/kg + low pressure hypoxia model group (LH-L + HACE group), the *ligustrazine hydrochloride* 100 mg/kg + low pressure hypoxia model group (LH-H + HACE group), and the dexamethasone 4 mg/kg + low pressure hypoxia model group (Dex + HACE group). Dexamethasone was chosen as the positive control agent for this investigation due to its efficacy in treating hypoxic hypobaria and the available literature [66]. According to the method provided by the supplier, LH and Dex were dissolved in 5% dimethyl sulfone (DMSO) until clarified, and then 40% polyethylene glycol 300 (PEG300), 50% double steaming water, and 5% Tween 80 (Tween 80) were added for intraperitoneal injection (i.p.) once a day for three days. The NC and HACE groups were administered a certain volume of the aforementioned solvent intraperitoneally.

In the second part of the investigation, we used the NRF2 inhibitor ML385 to evaluate LH’s regulation mechanism on the NRF2 signaling pathway [67]. Forty rats were randomly divided into four groups of ten each: NC group, HACE group, LH-H + HACE group, and *ligustrazine hydrochloride* 100 mg/kg + ML385 30 mg/kg + low pressure hypoxia model group (LH-H + ML385 + HACE group). ML385 dissolves in the same way as LH. To determine whether LH could prevent hypoxic-induced iron sag in hypoxic rats, rats in the LH-H + ML385 + HACE group were intraperitoneally injected with ML385 30 min before administration once a day for 3 days.

The selection of all the above doses and routes of administration was referred to previous studies [20,68]. One hour after the end of administration on the third day, except for the NC group, they continued to live in a normal pressure and oxygen environment. Treatment groups were placed in a low-pressure anoxic chamber to establish the HACE model.

#### 4.3.2. Establishment of Animal HACE Model

**Figure 11:**
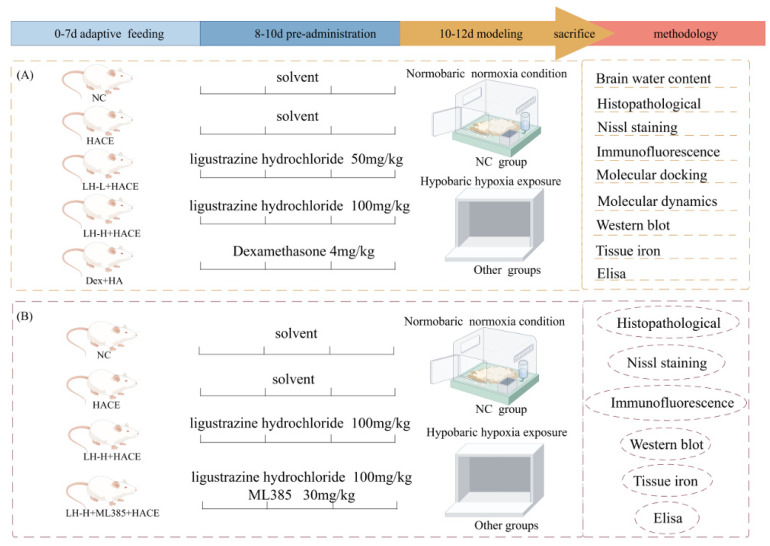
Schematic diagram of the experimental program of the research. (**A**) Study on the pharmacodynamics and ferroptosis phenotype of LH in HACE rats; (**B**) The mechanism study of LH on HACE rats through the NRF2 signaling pathway.

Following prior experimental research [69], rats were exposed to a simulated high-altitude environment in a hypoxic animal test chamber (FLYDWC50-II C, Avic Guizhou Fenglei Aviation Armament Co., Ltd., Guiyang, China) for 1 h after the final dose. The rats were placed in an anoxic chamber at an altitude of 6000 m to simulate the plateau environment and maintained for 48 h. The rats’ food and water intake were not restricted during modeling. In addition, a certain amount of sodium lime and anhydrous calcium chloride was added to the cabin to prevent excessive CO_2_ concentration and excessive humidity. After the modeling process, the rats were quickly removed, and their body weights were recorded; they were then anesthetized with a certain amount of sodium pentobarbital, and blood and brain tissue were collected. The experimental flow is shown in Figure 11.

### 4.4. Body Weight

Throughout the duration of the experiment, the physiological status of the rats was meticulously monitored on a daily basis, and their body weights were systematically recorded. A statistical analysis of their weight was conducted on days 1, 4, 7, 10, and 12.

### 4.5. Cerebral Water Content

Following the end of the modeling process, the rats were humanely euthanized by administering pentobarbital sodium intraperitoneally. Subsequently, the left hemisphere of the brain tissue was extracted, weighed with a high-precision electronic balance, recorded as wet weight, quickly transferred to an oven preheated to 80 °C for 48 h until its constant weight, and weighed again as dry weight. The formula [(wet weight − dry weight)/wet weight] × 100% was used to determine cerebral water content, indicating the extent of brain edema [70].

### 4.6. HE and Nissl Staining

After sampling, the right hemisphere of the rat brain tissue was rapidly fixed with 4% universal paraformaldehyde solution. The fixed brain tissue underwent dehydration using ethanol gradients, was cleared with xylene, and was then embedded in paraffin for sectioning. After brain sections were dewaxed in water, they were treated with hematoxylin-eosin and 1% toluidine blue for HE and Nissl staining, respectively, and finally sealed [71]. A Pannoramic 250 digital slide scanner by 3DHISTECH, Hungary (Pannoramic 250Flash) was used for acquiring images of the sections.

### 4.7. Inflammation and Oxidative Stress

Following rat modeling, blood was collected from the abdominal aorta using a lithium-heparin tube, and serum was extracted by centrifuging the tube at 3000 rpm for 15 min at 4 °C for subsequent analysis. Serum concentrations of IL-6, IL-1β, TNF-α, MDA, GSH, and SOD were measured using an ELISA kit according to the manufacturer’s instructions.

### 4.8. Tissue Iron

According to the total iron ion test kit provided by the manufacturer, brain tissue was mixed with total iron assay buffer at a ratio of 9:1 and then homogenized for lysis. Post-lysis, the tissue was centrifuged at 1000 g for 15 min at 4 °C, and the supernatant was collected for total iron ion measurement. The tissue sample’s total ferrous ion concentration (μmol/kg fresh weight) was determined using the formula: (sample group value − blank group value)/(standard group value − blank group value) × standard total ferrous ion concentration (μmol/L) × sample dilution × sample volume (L)/sample mass (kg).

### 4.9. Immunofluorescence Staining

The brain tissue was fixed, embedded, and sliced. Initially, paraffin sections were treated with an eco-friendly dewaxing solution and anhydrous ethanol, followed by a rinse with distilled water. To minimize endogenous peroxidase activity, samples were incubated in a 3% H_2_O_2_ solution at room temperature for 30 min and subsequently blocked with 3% BSA. Following the removal of the blocking solution, primary antibodies NRF2 and GPX4 were introduced and incubated overnight at 4 °C. Next, the sections were washed and dried by shaking. The secondary antibody was subsequently added and incubated at room temperature for 50 min. After washing and drying again, cell nuclei were re-stained with DAPI staining solution and incubated in the dark for 10 min. The sections were sealed with an anti-fluorescence quenching agent, and images were captured using a Leica DM6B microscope (Beijing Prisys Instruments Co., Ltd.) [72].

### 4.10. Molecular Docking

The PDB database [73] supplied the crystal structure of NRF2 protein (PDB ID: 7x5e) for connection. The 3D structure of the small molecule LH was obtained from the PubChem database and underwent energy minimization using the MMFF94 force field.

In this research, molecular docking was done using AutoDock Vina 1.2.3 [74]. Prior to docking, PyMOL 2.5.5 was employed to prepare the receptor protein by eliminating water, salt ions, and small molecules. Subsequently, the docking box was configured to encompass the entire egg white. ADFRsuite 1.0 was used to convert small molecules and receptor proteins to PDBQT format for docking [75]. The global search detail for interconnection was configured to 32, while other parameters were kept at their default settings. The docking conformation with the highest score was selected as the binding conformation for further molecular dynamics simulations.

### 4.11. Molecular Dynamics Simulation

Using the initial structure, AMBER 22 software simulated the molecular dynamics of a small molecule-protein complex at the atomic level [76]. Prior to simulation, the small molecule’s charge was determined using the antechamber module and the Hartree–Fock (HF) SCF/6-31G* method in Gaussian 09 software [77,78]. The GAFF2 force field was applied to small molecules, while ff14SB was used for proteins. Hydrogen atoms were incorporated using the LEaP module, and a truncated octahedral TIP3P solvent box was positioned 10 Å from the structure. Na^+^/Cl^−^ ions were added to neutralize the charge and create topology and parameter files for simulation [79,80].

Prior to simulation with AMBER 22 software, the system underwent energy optimization using 2500 steps of the steepest descent method followed by 2500 steps of the conjugate gradient method. Subsequently, the system was heated from 0 K to 298.15 K over 200 ps at a constant heating rate and fixed volume. Next, a 500 ps NVT ensemble simulation was run at 298.15 K to evenly distribute solvent molecules. After that, a 500 ps NPT equilibrium simulation was carried out, and finally, a 100 ns NPT ensemble simulation with periodic boundary conditions was executed. During simulation, a 10 Å cutoff was applied to non-bonded interactions [81]. The Particle Mesh Ewald technique was employed for handling long-range electrostatic interactions, and the SHAKE algorithm was utilized to constrain hydrogen bond lengths [82]. The Langevin algorithm was employed to control temperature (collision frequency γ = 2 ps^−1^) [83]. With a system pressure of 1 atm, the integration step was set to 2 fs with trajectories saved every 10 ps for analysis.

### 4.12. MM/GBSA Combined Free Energy Calculation

The MM/GBSA method can be used to calculate the binding free energy between proteins and ligands across all systems [84,85,86,87]. To avoid compromising calculation accuracy, the study employed the MD trajectory between 90 and 100 nanoseconds [85]. The following is the formula: ΔG_bind_ = ΔG_complex_ − (ΔG_receptor_ + ΔG_ligand_) = ΔE_internal_ + ΔE_VDW_ + ΔE_elec_ + ΔG_GB_ + ΔG_SA_. The formula includes internal energy, van der Waals, and electrostatic interactions, where internal energy consists of bond, angle, and torsion energies. ΔG_GB_ represents the polar free energy of solvation, while ΔG_GA_ accounts for the non-polar free energy. The GB model (*igb* = 2) was introduced by Nguyen et al. [88]. The non-polar solvation free energy (ΔGSA) is determined by the product of the surface tension (γ) and the solvent-accessible surface area (SA), expressed as ΔG_SA_ = 0.0072 × ΔSASA. Due to their resource demands and low precision, entropy changes are excluded from this study.

### 4.13. Western Blot

Brain tissue was weighed in a certain amount for total protein extraction, with the addition of the appropriate RIPA cracking buffer, and 50× Cocktail protease inhibitor, phosphatase inhibitor liquid A, liquid B, and PMSF were added, respectively. After grinding treatment in a high-throughput tissue grinder, the brain tissue was centrifugated at 12,000 rpm and 4 °C at a high speed for 15 min. The protein concentration in the supernatant was measured using the BCA protein assay kit (Thermo Fisher Scientific China website: 23227). Proteins were separated via SDS-PAGE and transfered to a PVDF membrane. The film was incubated in 5% skim milk at room temperature for 2 h, followed by three 10 min washes with TBST. Primary antibodies used include VEGF (1:3000), AQP4 (1:10,000), HIF-1α (1:500), NRF2 (1:500), GPX4 (1:1000), SLC7A11 (1:1000), FTH1 (1:1000), and TFRC (1:1000). These primary antibodies were incubated at 4 °C overnight, washed 3 times with TBST, and incubated at RT for 1 h with secondary antibodies at 1:10,000 dilution. Following three washes, proteins were detected using an enhanced chemiluminescence kit and analyzed with a Bio-Rad image analysis system.

### 4.14. Statistical Analysis

Data analysis and plotting were performed using GraphPad Prism 9.5 software (San Diego, CA, USA). Results are expressed as mean ± standard error. A one-way ANOVA was conducted for data with a normal distribution, while the Kruskal–Wallis test was utilized for data that did not follow a normal distribution. A *p*-value less than 0.05 was deemed statistically significant.

## 5. Conclusions

This study demonstrated that LH plays a crucial role in preventing HACE by mitigating oxidative stress and ferroptosis induced by high-altitude hypoxia through the activation of the NRF2 signaling pathway, thereby offering protection in HACE rats. The experimental findings validate the antioxidant properties of the natural compound LH, suggesting its potential to mitigate oxidative stress induced by high-altitude hypoxia. These findings offer a scientific foundation for LH in preventing HACE and present new opportunities for future natural drug development.

## Figures and Tables

**Table 1:** The binding affinity scores (kcal/mol) of the complexes.

Target Name	Ligand_name	Docking_score
7x5e-nrf2	ligustrazine hydrochloride	−4.083

**Table 2:** MM/GBSA (kcal/mol) predicted free energy binding and energy components.

System Name	ΔEvdw	ΔEelec	ΔGGB	ΔGSA	ΔGbind
NRF2/LH	−7.98 ± 4.14	−0.23 ± 1.45	5.46 ± 3.37	−0.97 ± 0.54	−3.73 ± 2.13

## Data Availability

The data used to support the findings of this study are included within the article.

## Abbreviations

The following abbreviations are used in this manuscript:

HACE	high-altitude cerebral edema
AMS	acute mountain sickness
HAPE	high-altitude pulmonary edema
MDA	malondialdehyde
GPX4	glutathione peroxidase 4
NRF2	nuclear factor erythroid 2-related factor 2
HIF-1α	hypoxia-inducible factor-1α
AQP4	aquaporin 4
VEGF	vascular endothelial growth factor
GSH	glutathione
SOD	superoxide dismutase
GSH-Px	glutathione peroxidase
FTH1	ferritin heavy chain 1
LH	ligustrazine hydrochloride
TFRC	transferrin receptor
SA	surface area
KEAP1	Kelch-like epichlorohydrin-related proteins
IL-1β	interleukin-1β
IL-6	interleukin-6
RMSD	root mean square deviation
RMSF	root mean square fluctuation
